# Geoffrey: An Automated Schedule System on a Social Robot for the Intellectually Challenged

**DOI:** 10.1155/2018/4350272

**Published:** 2018-12-02

**Authors:** Edmanuel Cruz, Félix Escalona, Zuria Bauer, Miguel Cazorla, José García-Rodríguez, Ester Martinez-Martin, José Carlos Rangel, Francisco Gomez-Donoso

**Affiliations:** ^1^Institute for Computer Research, University of Alicante, P. O. Box 99. 03080, Alicante, Spain; ^2^RobotSIS, Universidad Tecnológica de Panamá, Panama City, Panama

## Abstract

The accelerated growth of the percentage of elder people and persons with brain injury-related conditions and who are intellectually challenged are some of the main concerns of the developed countries. These persons often require special cares and even almost permanent overseers that help them to carry out diary tasks. With this issue in mind, we propose an automated schedule system which is deployed on a social robot. The robot keeps track of the tasks that the patient has to fulfill in a diary basis. When a task is triggered, the robot guides the patient through its completion. The system is also able to detect if the steps are being properly carried out or not, issuing alerts in that case. To do so, an ensemble of deep learning techniques is used. The schedule is customizable by the carers and authorized relatives. Our system could enhance the quality of life of the patients and improve their self-autonomy. The experimentation, which was supervised by the ADACEA foundation, validates the achievement of these goals.

## 1. Introduction

The increment of life expectancy and the low mortality rates in developed countries are bringing an accelerated growth of the percentage of elder people. As remarked by the United Nations (https://population.un.org/ProfilesOfAgeing2017/index.html), currently, 12.74% of the population is above the threshold of 65-year-old, but as for 2050, this is expected to grow up to 27.04% of the global population. This quick population aging is one of the principal concerns of developed countries and one of the priority lines of research. In addition to the elder people, thousands of people worldwide are affected by brain-related injuries nowadays. These diseases can be caused by different situations such as trauma, accident, or even by genetic affections. The alterations that can arise after an acquired brain injury with increasing age include the loss of intellectual abilities of different severity. These disabilities interfere with social or occupational functioning, memory, or abstract thinking disorders, the inability to find similarities and differences between related words, or the difficulty to perform common domestic tasks, among others. Specifically, one of the most common conditions is the omission of the natural order to perform a task. For instance, the affected people for this condition would brush their teeth before applying the toothpaste or take the toothbrush but eventually forget what comes next.

One of the worst outcomes of being part of these collectives is the reduced personal autonomy. Elder and intellectually challenged people often require the special attention of a therapist that helps them to perform diary tasks such as tying the shoelaces, taking a shower, or having the meals.

In this context, we propose the implementation of an automated schedule system on a social robot that would assist the patients in their daily tasks at home. The system will notify the programmed tasks to the patients on a scheduled time and will also help to achieve the actions by walking the patient through them. It will detect if the user is doing well and give feedback if not.

The implementation of the proposed system would improve the personal autonomy and enhance the quality of life of elderly and intellectually challenged people.

So, the main contributions of this work are

- A programmable schedule system deployed on a social robot
- An integration of different methods in order to monitor if the patient is performing the actions he is intended to perform
- A navigation system which consists of a mix of semantic localization methods and the traditional SLAM

The rest of the paper is structured as follows. First, the state of the art is reviewed in Section 2. In Section 3, our proposal is thoroughly explained by giving an overview and then focusing on each piece of the system. Then, the experimentation is detailed in Section 4. Finally, the conclusions and limitations of this work are discussed in Section 5.

## 2. Related Works

The growing number of elder people is being increasingly important to promote the role and technological advance of social and assistive robotics. Speaking about Social Robotics creates the necessity to actually define what a social robot is. According to [1], it is defined as *“A physical entity embodied in a complex, dynamic, and social environment sufficiently empowered to behave in a manner conducive to its own goals and those of its community*.*”*

In this specific field, we have to divide the different works based on their application. There are projects for assistance in medical environments [2, 3], whilst some others are focused on the emotional and cognitive tasks [4, 5], and there also are projects for a social assistive task in different environments. This state of art is focused on this last issue.

In 1998, PAM-AID (Personal Adaptive Mobility Aid) [6] was created. This system aims to provide both physical support during walking and obstacle avoidance. It used sonar, infrared proximity sensors, and bumpers switches to get information from the environment.

One-year later, the system proposed in [7] emerged. It described the implementation of a control architecture for robots designed to combine a manipulation task with a motion controller that used the operational space formulation to define and implement arm trajectories and object manipulation.

The same year appeared the first so-called intelligent wheelchair [8]. The system provided different functions: from fully autonomous navigation in an unknown crowded environment to partially autonomous local maneuvers. Two years later, on the same topic, the study [9] was created, which described the mounting of a robotic arm to a powered wheelchair to assist disabled users in daily activities.

In 2003, three different types of work appeared. First, Falcone et al. [10] describe the efforts to design, prototype, and test a low-cost, highly competent personal rover for the domestic environment. Then, Pineau et al. [11] describe a mobile robotic assistant developed to assist elderly individuals with mild cognitive and physical impairments, as well as support nurses in their daily activities. They used three software modules: an automated reminder system, people tracking and detection system, and a high-level robot controller. Finally, Pollack et al. [12] use AI techniques to model an individual's daily plans, observe and reason about the execution, and make decisions about whether and when it is most appropriate to issue reminders.

Years later, it appeared the PAMM [13] project, which is a system for support and guidance. The PAMM detects and maneuvers away from obstacles, and it uses an upward looking camera for localization and also can communicate with a central computer. The central computer provides the system with a map of the facility including the location. In turn, the system provides the central computer with the user's location, health status, and requests.

In 2010, the system described in [14] was proposed. It aims at designing a socially assistive robot to monitor the performance of the user during a seated arm exercise scenario, and the main purpose was to provide motivation to the user to complete the task and to improve performance.

Also this year, “the home exploring robotic butler” (HERB) [15] was published. It can efficiently perform mapping tasks, searching, and navigation through indoor environments, recognize and localize several common household objects, and perform complex manipulation tasks.

In 2011, the system described in [16] appeared. It was an indoor mobile robot for taking care of the elderly. It has a human physiological parameters monitor system, which can take care up to six nursed persons by using a variety of sensors.

The ASIBOT [17] was published one-year later. It helps users to perform a variety of tasks in common living environments. The robot is able to autonomously climb from one surface to another, fixing itself to the best place to perform each task. It also can be attached to a wheelchair, giving the user the possibility to move along with it as a bundle.

A new iteration of the aforementioned HERB [15] system emerged in 2012. The HERB 2.0 [18] consists of a two-handed mobile manipulator that can perform useful tasks for and with people in human environments.

In 2014, it was created as a multiuser human-robot interaction (HRI) [19] system architecture to allow the social robot Tangy to autonomously plan, schedule, and facilitate multiuser activities that consider the users' necessities. During the activities, the robot was able to interact with a group of users providing group-based and individualized assistance based on the needs of the individual. The same year, the robotic nursing assistant (RoNA) was created [20], which would assist nurses while performing intensive tasks and prevent musculoskeletal injuries among health care workers.

The robotic exercise tutor [21] was published in 2017. The created humanoid robot learns exercise routines from a human trainer and performs them in front of the elderly. Its main task was to monitor the performance of the patients and provide feedback.

In 2018, deep learning (DL) algorithms were introduced into assistive robot systems. For instance, a remote health care system based on moving robots intended for the elderly at home was proposed in [22]. The robot is able to perform different kind of tasks. The user can control the robot and call it using voice commands or with a phone, and it also performs object detection and pose estimation. It even can monitor the posture of the elderly and collect and transmit the data recorded by a set of sensors connected to the robot to the cloud for further analysis. On its behalf, PHAROS, a robotic assistant for assisting elderly in their daily physical activities at home, was proposed in [23]. This interactive robot platform is divided into two modules: the recommender (recommends activities at a scheduled time) and the human exercise recogniser (as its name implies, it is the identifier of the human pose). This system works in real time and uses deep learning methods to properly recognize the performed physical exercises.

## 3. Proposal

In this work, we propose a robotic system for monitoring and interacting with people affected by cognitive disabilities and elder people. The system will guide the patient through their daily tasks helping, guiding, and encouraging them to follow a preset schedule. The ultimate goal of the proposed system is to improve the quality of life of dependent people and their self autonomy.

The system is composed of a programmable schedule and a contextual schedule. The programmable schedule is composed of a list of tasks that the patient must perform at a certain time. This kind of tasks could be delayed if the patient is authorized to do so. On the contrary, the contextual schedule is composed of a list of tasks that the patient is authorized to perform on demand, and it will depend on the room the user is located.

**Figure 1:**
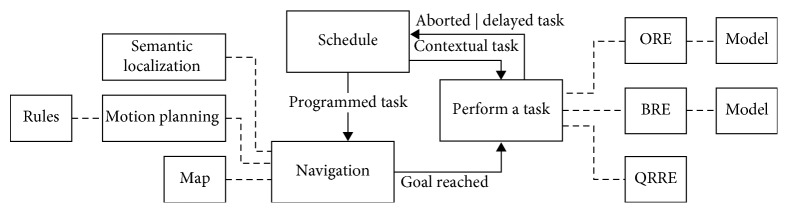
Diagram of the proposal.

As shown in Figure 1, when the patient is notified to complete a task of the programmable schedule, the patient could try to complete it now or delay it. If the patient chooses to perform it upon requirement, the robot will first guide the patient to the appropriate room, and then, it will provide instructions in order to perform the required task. If the patient delayed a task, the system will ask him to complete it later.

Whether the tasks come from the programmed or the contextual schedule, each one is composed of a list of actions, which are goals the patient must accomplish to complete a task. The tasks are assigned and setup by the therapist in charge of the patient or authorized relatives. Currently, our system comprehends four main different types of actions: object recognition, behavior recognition, QR recognition, and spend time action. As aforementioned, the person in charge of the patient can combine these actions to construct high-level tasks. For instance, the task “pour a glass of water” would be composed of three different actions. First, the robot would ask the patient to get a glass and show it, and then, it would ask to show the bottle of water. Both actions would use the object recognition engine in order to detect a glass and a bottle. Finally, the behavior recognition engine would be setup to detect the actual action of pouring. If the user consistently fails to complete an action, the task is automatically aborted (and delayed if it was configured like so). In addition, the carers could be notified of this event if they choose to. Similarly, the task “take the medicine A” would involve two tasks. First, the robot would ask to show the medicine A package, which would be labeled with a QR code. The action in this case would use the QR recognition engine to check if the object showed by the patient is correct. Then, it would spend some time idle waiting for the patient to take the medicines by making use of a spend time action. Through the achievement of the tasks, the robot is continually providing both visual and speech feedback to inform and encourage the patient upon the completion of the task.

It is worth noting that the system is intended to be deployed in a social robot that will follow the patient wherever he goes.

### 3.1. The Social Robot

**Figure 2:**
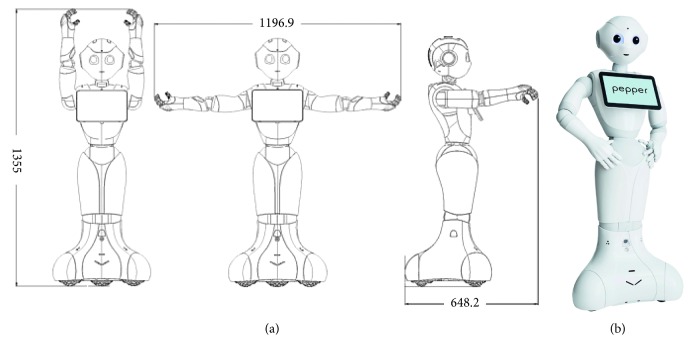
The proposed system is intended to be deployed on a Pepper robot, but it can be deployed in any robot that meets the requirements of our system. (a) Some physical features of the Pepper robot, whilst the (b) depicts its visual appearance.

A Pepper robot was chosen in order to develop the proposed system. As shown in Figure 2, the Pepper robot is a human-shaped robot manufactured by Softbank Robotics. It features a variety of sensors such as radar, laser, RGB-D cameras, and microphones among others.

We adopted it for two main reasons: first, its appearance is familiar and engaging so the users feel comfortable when interacting with it. On the contrary, its features suit perfectly the requirements of our proposal. The requirements are a mobile base and size that allow the robot to easily move in indoor environments in order to guide the patient to the desired room and to follow him and a microphone that enables speech and voice recognition capabilities. They provide a natural interaction mechanism; a tablet to display relevant information and feedback and to offer an alternative interaction method; a camera to monitor the patient and detect if the given directions are being followed; and a front laser which is in charge of detecting obstacles and provide local localization capabilities.

It is worth noting that any other robot that meets the aforementioned requirements can be used to deploy the proposed system.

### 3.2. Object Recognition Engine

As aforementioned, there exist actions in the tasks that are about detecting a certain object, for instance, the detection of a glass in the task, “pour a glass of water.”

To do so, we implemented an object recognition engine (ORE). The ORE is based on the InceptionResNetV2 [24] architecture. This DL-based method is proven to provide one of the lowest Top-1 and Top-5 errors on the ImageNet ILSVRC 2012 [25] challenge. This architecture takes advantage of the inception concept and residual connections in order to obtain high accuracy rates while maintaining the computational cost at bay.

We adopted a classic convolutional neural network (CNN) scheme over region-based convolutional neural network (R-CNN) architectures to avoid false detections. For instance, when the robot asks for a glass, it is intended that the patient looked for the object and held it in front of the robot. Nonetheless, if the patient is in the kitchen, it is likely that there were glasses on the counter or on a table that a R-CNN would detect. Given this case, it is difficult to differentiate whether the patient is holding the object or it appeared in the background of the scene. On the contrary, taking advantage of a classic CNN approach, the object is correctly detected if only the object is depicted in the input image. This way, we force the patient to look for the desired object and to show it on purpose to the robot.

When the ORE is used by the system, it first captures an image using the camera of the robot. The image is classified by the ORE and if the returned label matches the desired object, the action is considered fulfilled. If not, the process is repeated up to a preestablished number of trials. If the object is not correctly detected, the whole task is aborted. We consider detection if any of the scores assigned to a label is above a certain threshold. If no label is above this threshold, no object is considered as detected.

Note that the ORE must be used when high generalization capabilities are required. As aforementioned, when asking for a glass, any glass would work. So, we need the classifier to recognize any kind of glass.

### 3.3. Behavior Recognition Engine

**Figure 3:**
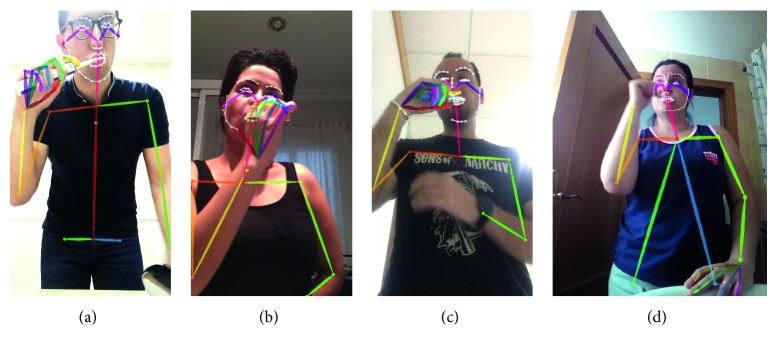
Human skeleton detection of several people in different bathrooms by using Openpose [26, 27, 28].

Once the necessary objects to carry out the requested action are identified, the next step is to properly recognize the user's behavior. For that, the first step is to robustly detect the person(s) within the image. However, this is not a straightforward task due to the required generality of the system. So, the designed behavior recognition engine (BRE) should be able to properly recognize the user's behaviors in different rooms and in different houses. Therefore, background subtraction techniques are discarded. In addition, no requirements about patient's appearance can be established. As a consequence, an abstraction mechanism is required. In particular, the skeleton-based representation Openpose [26, 27, 28] is used. Basically, this two-branch multistage convolutional neural network (CNN) outputs a 18-keypoint body skeleton for all the people in the image, independent on the background or the person, as illustrated in Figure 3.

**Figure 4:**
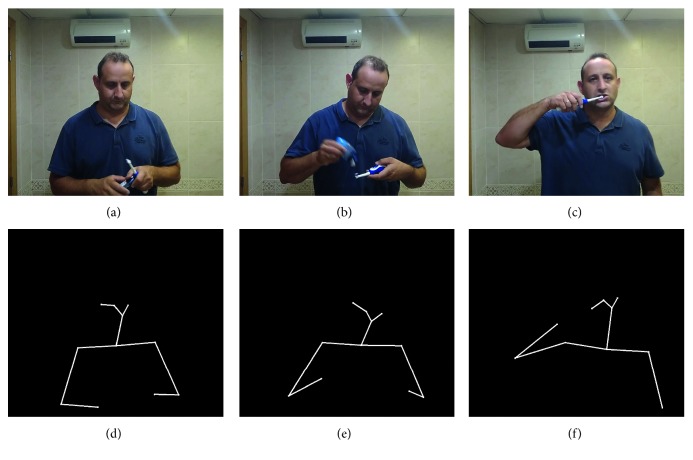
Human poses representing the brushing teeth behavior. (a)–(c) depicts images as captured by the robot, whilst (d)–(f) shows the corresponding estimated skeleton.

From this body keypoint information, a new image focused on the human skeletons is generated. In this way, the behavior recognition is reduced to a human pose classification. That is, each behavior can be defined as a sequence of several human poses such that their classification allows the system to recognize the represented behavior (see Figure 4 for example).

As a classification problem, a CNN architecture could fit. Particularly, in this paper, a *ResNet50* [29] was used. *ResNet50* is a deep residual network of 50 layers that have been trained to the task at hand.

**Figure 5:**
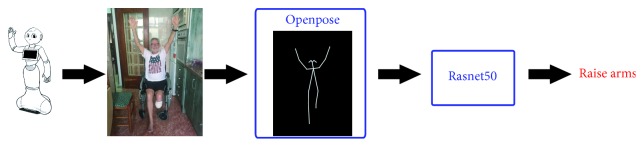
Flowchart corresponding to the implemented behavior recognition engine (BRE): the top Pepper's RGB camera captures an image that is processed by Openpose to get a skeleton-focused image. This image feeds the trained ResNet50 to properly recognize the observed behavior.

Thus, the BRE flowchart can be described as follows: the robot RGB camera takes an image, from which a 224 × 224 × 3 image with human skeletons is generated. This skeleton-based image feeds the pretrained *ResNet50* that outputs the observed behavior (Figure 5).

### 3.4. QR Recognition Engine

There are actions in the tasks that require a much finer detection, meaning, a certain instance of an object. For example, in the action “show the medicine A,” the robot is asking for a particular medicine. The ORE cannot be used in this case because the requested object is very specific, and its main goal is to provide high generalization capabilities. So, the QR recognition engine (QRRE) is intended to be used when the required specificity of the requested object is critical.

To do so, we implemented a QR code detector. The QRRE is based on Zbar [30], which is an open-source barcode and QR codes scanner. As expected, the objects must be manually tagged with the correspondent QR code, so the QRRE could be used to recognize them. We chose this method over the traditional object recognition pipeline [31, 32, 33, 34] because it is much faster and reliable.

When the QRRE is used by the system, first it captures an image using the camera of the robot. The image is fed to the QRRE, and if the returned label matches the desired object, the action is considered fulfilled. If no object is correctly detected within the preestablished number of trials, the whole task is aborted.

### 3.5. Speech Recognition Capabilities

In order to allow a natural way of interaction with the robot, our system takes advantage of the built-in speech recognition capabilities offered by the Pepper robot. The speech recognition engine is provided by Nuance [35], which is a company experienced in this area. This company is in charge of developing top-tier commercial speech recognition software.

The speech recognition engine is able to identify predefined words and statements configured by the user. We adopted it as one of the main interaction methods our system offers and is used when the robot asks a question. For instance, the robot expects a “yes” or “ok” or a “no” when it asks the user if he would like to perform a programmed task now. The tablet of the robot displays a “listening” message, and the robot makes an acoustic alert whenever the speech recognition engine is expecting an answer from the patient, so he would know that he can interact with the robot using this method.

### 3.6. Semantic Localization System

The aim of the semantic localization system (SLS) is to compute the location at the semantic level. To do this, the work based the SLS on was presented [36]. In this work, an optimal methodology for a mobile robot to adapt its knowledge to new environments was proposed. This module works in the following way.

First, the robot captures images of the environment and tries to classify them using an initial pretrained model. The images used to train this model come from unknown and different homes. As it is deployed in a new environment, it is likely that the system obtains low accuracy rates. This is due to the different visual features of the new environment and the environments in which the model was trained in the first place. In this case, we can provide information to the robot to collect data and reidentify the locations. In case the category provided by the user is not considered so far by the model, it will be added as a new category. This way, the robot can easily increase and fit its knowledge to the new environment.

It is worth noting that the model fitting of the SLS is performed before the robot is actually deployed, so it can precisely localize itself once deployed without the interference of the patient.

**Figure 6:**
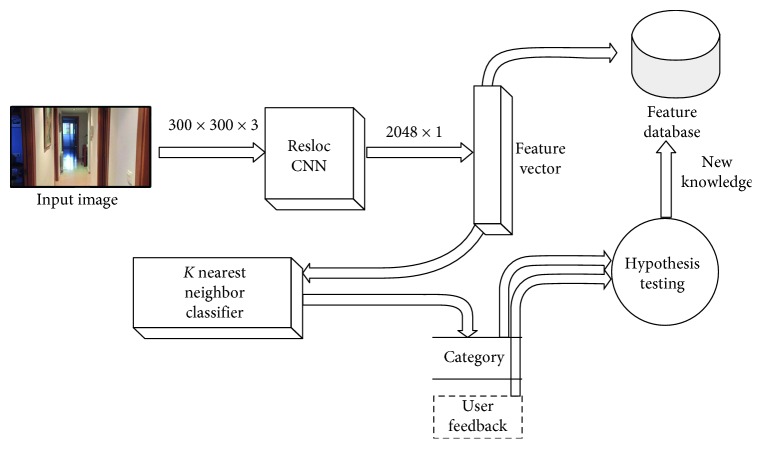
This architecture uses the features of a ResLoc CNN with a vector of 2,048 features as the output. The training samples are forwarded to the ResLoc CNN in order to extract their feature vectors. The feature vectors construct the model of a KNN classifier.

To achieve this goal, we use the architecture showed in Figure 6. This works as follows: an input image is forwarded to the ResLoc CNN architecture. This is a classic CNN architecture which lasts until the fully convolutional layer was removed, so the output is the visual features descriptor for the input image. As a result, the output of ResLoc CNN is a 2,048 dimensions feature vector.

The visual features and the correspondent categories of each image of the training dataset are extracted using the ResLoc CNN part of the architecture and inserted on the features database. This features database is a model that stores the learned data, which are the features of the training samples. This model is used during the inference stage.

On the inference stage, the unknown image is forwarded to ResLoc CNN in order to extract the visual feature vector. Then, a *K*-nearest neighbors (KNN) classifier performs a query on the feature database using the recently computed feature vector. Next, a polling is carried out among the categories of the neighbors, and the most voted category is returned as the final classification of the unknown image.

The performance of the KNN is highly dependent on the *k* parameter (number of neighbors). Experimentation on this matter is carried out to set the best performing *k*. We used the Annoy [37] implementation of the (approximate) KNN classifier.

Then, the model is specialized even more with samples that come for the actual house in which it is deployed. The new samples are inserted only if the localization fails in a certain room.

As a consequence of this method, the SLS is always updating its model to prevent loosing performance, thus, adapting it as the time goes past. This is specially useful as the appearance of the environment is inevitably going to change. For instance, the furniture is eventually being changed or rearranged, the walls are being painted of another color, or the home appliances are being replaced.

### 3.7. Motion Planning System

When a new location goal is determined as a consequence of triggering a task that must be performed on another room, we need a system that calculates the path from the current room to the target. This task can be done using a simplified map of the environment and an expert system that computes all the paths between the actual location room and the destination room using some connection rules and facts. We named this system the motion planning system (MPS).

The map is modeled as a graph where the nodes are rooms, doors, and intersections between location access, and the edges are the connection between these places. These connections have an associated direction of movement for the transition (north, east, south, and west) and a travel cost that represents the distance between the nodes. Also, we define the transition matrix between node types, where we represent the action that must be performed going from the node type *A* to *B*, as shown in Table 1. Cross type is a node outside a tagged room where various ways join. Interior type is a node inside a tagged room where various ways join. The actions are cross the door (cd), follow the corridor (fc), and ND (not defined).

The expert system that computes the paths between nodes has been developed in Prolog, a logic programming language that provides great tools for this kind of task, such as declarative rules and unification (for restrictions management) and backtracking (for graph exploration).

The knowledge is divided into two separated files: facts and rules. The facts are specific to the concrete environment that is being modeled. They contain the definition of the nodes with their types (rooms, doors, cross, and interiors), the connection between nodes with the direction and the associated movement cost, and a dynamic predicate that indicates if a door is closed, which can be modified at runtime with the information provided by the robot sensors.

The rules are common to every environment modeled in this way. They check the connectivity between the nodes (direct or indirect) and calculate the path, set of directions, set of actions, and the cost from node *A* to *B*. In facts files, the direct connectivity between nodes is represented only in one direction, so we have defined rules that allow the reverse computation of this connectivity, looking for connections from node *A* to *B* and from node *B* to *A* reversing the direction of the movement, as shown in Sourcecode 1. The same principle has been applied to the computation of the actions, as shown in Sourcecode 2. The predicate action/3 is the Prolog representation of the transition matrix shown in Table 1.

For the building of the paths between the nodes, we recursively search for those that are directly connected to the current one until we reach the final node. We have to notice that we are looking for paths without loops (trees), so we do not allow the repetition of any node in them. Without this restriction, the computation of this exploration would hang and enter an infinite loop.

Due to the flexibility of the Prolog module, we cannot only calculate the paths between the defined nodes *A* and *B,* but we can make much more queries, like discovering all the accessible nodes from every node, using the same facts and rules knowledge.

### 3.8. Navigation and Mapping

We also relied on the ROS framework for the complete control of the movement of the robot. This framework provides utilities to interact with the robot and the mapping and navigation methods we adopted for our system.

The task of moving the robot from its current location to another part of the house requires a list of waypoints, which correspond to the labels (one per room) that are used by the SLS and the MPS and the correspondent locations in the robot coordinate frame. When a task is triggered in a different location in which it is intended to be carried out, the robot guides the patient to the intended location. To do so, the robot localizes itself using the SLS. As a result, a semantic label is obtained. Then, the semantic label is looked up in the waypoints list. This way the robot is approximately localized. Next, the MPS is used to build a plan from the current location to the final destination. This plan is a list of waypoints, and the robot will try to reach one by one.

We relied in the *gmapping* ROS package to create the MPS waypoint list. This method reads the data provided by the laser and creates an occupancy grid map using it. The algorithms that comprehend *gmapping* are thoroughly explained in [38]. Despite being intended to be used by a laser sensor, we have not used the integrated laser sensors of Pepper because of the lack of resolution, so we adopted the depth image to fake laser readings, as stated in [39].

The output of this step is a static 2D map that defines the limits where the robot can move through the walls, the doors, and the architectural barriers, but not the moving obstacles. In this map, we define the pose of the waypoints of the MPS, so we can translate the semantic locations to physical positions.

Once we have build the map of the environment, we can load and use it to perform the navigation. Then, we need to determine the position and the orientation of the robot within the map every time it moves. To do this, we used the Monte Carlo localization implemented in the adaptive Monte Carlo localization (AMCL) ROS package, which is explained in [40]. This method samples a set of particles in each iteration that represents a set of probable current poses of the robot. It uses the information provided by the sensors to determine the validity of that prediction and concentrates the next predictions around the older most probably ones.

**Figure 7:**
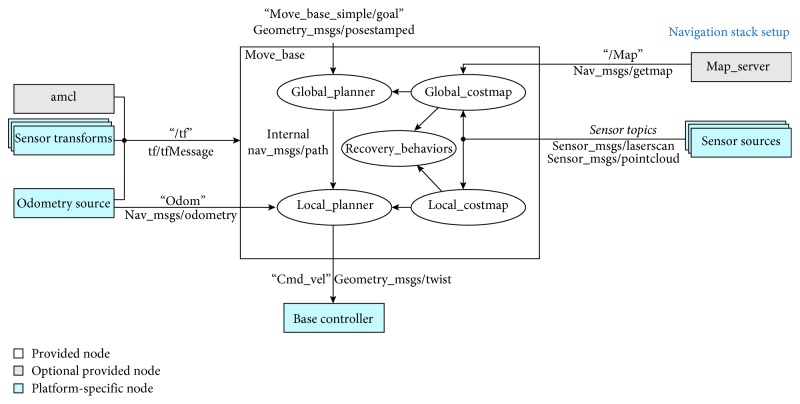
Global scheme of the navigation stack. Extracted from [41].

Finally, we have to resolve the path planning between the current pose of the robot and the target position and move the robot to the goal. This task is done using the ROS *move_base* package, one of the main elements in the ROS navigation stack. It receives a goal pose as input and then communicates with components such as the global and local planners, recovery behaviors, and costmaps and generates a velocity command for the base of the robot until it reaches the desired position. The components used by this node are explained in [41] and in the related links of that page (Figure 7).

Finally, it is worth noting that we used this mix of semantic localization and traditional mapping and navigation systems because the pure SLAM techniques tend to loose performance on the long term. It is very likely that the robot will not recover the localization once lost despite the accurate state-of-the-art SLAM methods. However, SLS provides an even more accurate localization method because it is based on visual features instead of laser features or odometry, which are often insufficient to provide a robust localization over time.

### 3.9. People Tracking and following Behavior

**Figure 8:**
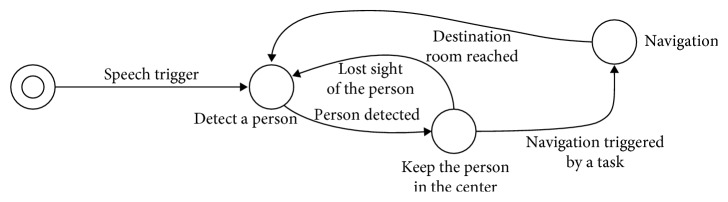
Finite states machine that models the people tracking and following behavior of the robot.

As aforementioned, the robot is intended to always stay by the patient in order to be noticed when it announces a scheduled task. To implement this behavior, we modeled it as a finite state machine as it is shown in Figure 8.

In the initial state, it starts by waiting for the patient to show in front of the robot and saying a customizable trigger statement. If the robot detects that statement using its speech recognition system explained in Section 3.5, then it tries to detect a person. To do so, we relied in YOLOv3-320 [42]. This is a region convolutional neural network architecture that is able to detect the position of the objects in the image plane, the label of those objects, and the correspondent detection score. This architecture achieved 0.51 mAP (measured over the intersection over union) over the test set of the COCO MS dataset. It is currently a state-of-the-art method on object detection and recognition providing a decent accuracy with low computation cost.

So, the robot uses its camera to forward the color data to this architecture in order to detect a person. If only one person is detected, the robot tries to maintain it in the center of its sight within a threshold by moving its base left or right. The robot also keeps a clear distance between itself and the patient of a customizable distance. This distance is computed using the front laser sensor of the robot. If the patient walks away, the robot must follow him by setting new goals to its navigation system, which is explained in Section 3.8, but always keeping the patient in the center of its sight and at the preset distance. It is worth noting that it will not move away when the patient approaches the robot. If the robot looses track of the person, it will be announced with a speech notification, and then, it will proceed to halt and try to detect a person one more time.

This behavior is kept until a programmed or contextual task is triggered, and navigation to a goal is required. In this case, the robot takes a role in which it is in charge of leading the patient to a destination room in order to perform the requested task. When the destination room is reached, the robot turns around (it assumes the patient is following it) and changes to the person detection role once again.

This simple yet effective tracking and the following system enables the robot to stay besides the patient at all times allowing a natural and fluent interaction.

## 4. Experimentation, Results, and Discussion

Before the deployment of our system in an actual scenario, the therapists and the persons in charge of the patient must define the tasks that the patient is able to perform. The tasks defined for this pilot experience are shown in Table 2. These tasks were suggested by therapists of ADACEA, which is a foundation for the acquired brain injured people.

**Figure 9:**
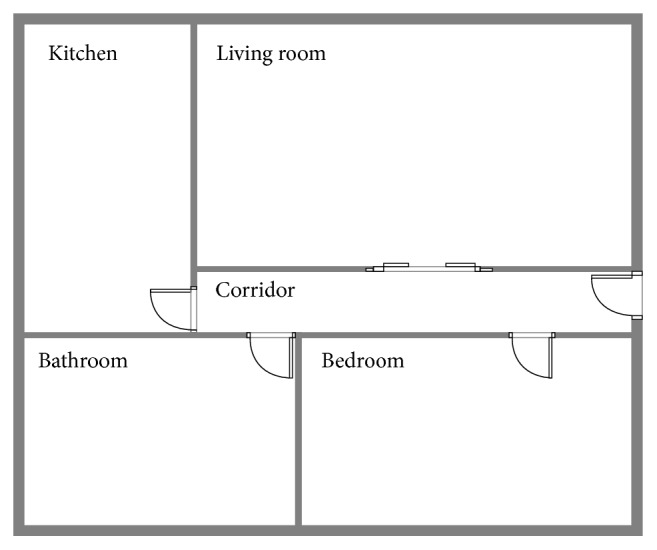
The actual plan of a test house.

Then, it is required to build the initial model of the SLS and the corresponding map for the MPS.  Figure 9 shows the plan of the test house with their rooms. Note that the navigation system will not use the full map but the waypoints in order to set the next navigation goal. The SLS subsystem will provide a good approximate localization for the navigation step.

The BRE and ORE models were also trained beforehand. Our approach assumes that this step is already done and it can use the aforementioned maps and trained models.

In the following subsections, we provide experimentation of each piece that compose the system.

It is also worth noting that some of the experiments involved actual patients and their homes, but in some others, the patient had to be simulated by fellow research mates. This is due to lack of authorization from the patients.

Finally, as the computation power of Pepper robot's integrated processor is quite limited, the computation of the ORE, the BRE, and the execution of the YOLO architecture are performed in an additional computer equipped with a Nvidia GTX 1080Ti GPU. The robot and the additional computer are interconnected using the ROS framework. The neural architectures were developed using the Darknet and Keras frameworks.

### 4.1. Object Recognition Engine Experimentation

**Figure 10:**
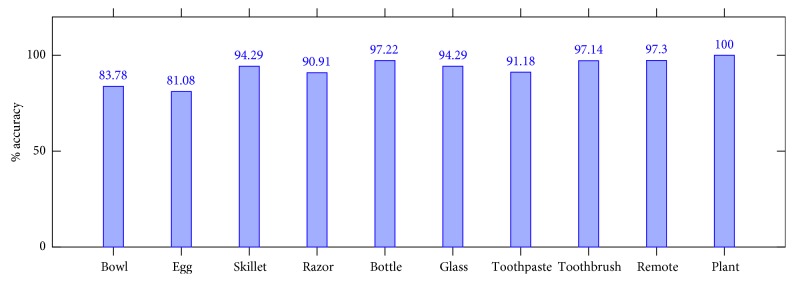
Accuracy per class distribution of the test split obtained by the object recognition engine.

**Figure 11:**
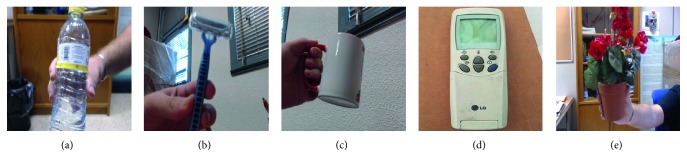
Samples of objects correctly detected by the object recognition engine. Note that these samples do not belong to any train, validation, or test split.

The architecture was trained ad-hoc for the ORE. In order to build the dataset, we downloaded the first 400 most relevant images with a public domain license from Google images for each object intended to be detected. These objects are those required by the tasks the patient must perform. So, the objects covered by the ORE are toothbrush, remote, bowl, toothpaste, bottle, egg, skillet, glass, and razor. The images were distributed in the training, validation, and test splits at 70%, 20%, and 10% each. The optimizer of choice was Adam, with a learning rate of 0.0001. The architecture was initialized with the ILSVRC 2012 model and trained for 10 epochs reaching a validation accuracy of 92.75% and a test accuracy of 92.61%. It is worth noting that the detection threshold was empirically set to 0.6.  Figure 10 depicts the accuracy per class of the test split.  Figure 11 depicts some samples of these objects correctly detected by the robot in the context of different task guidance.

### 4.2. Behavior Recognition Engine Experimentation

**Figure 12:**
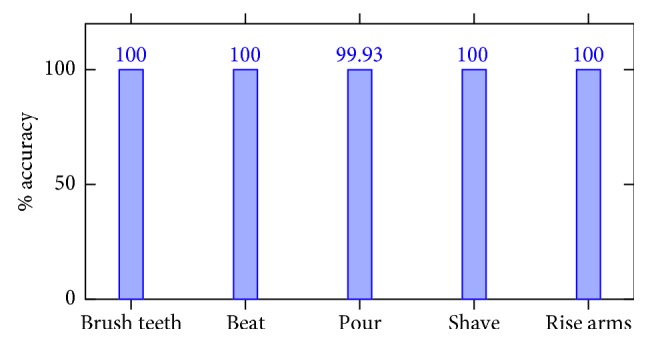
Accuracy corresponding to the test set of the behavior recognition engine after 150 epochs of training.

With the aim of evaluating the BRE's performance, several subjects (nine in total; four women and five men) were recorded carrying out the five considered behaviors (i.e., shave, pour, brush teeth, beat, and rise arms) in different scenarios. So, all the video sequences captured by the top Pepper's RGB camera were divided into frames and manually labeled with the observed behavior. Then, these images were processed by Openpose [26, 27, 28]. After that, data augmentation was applied in order to properly identify left- and right-handed behaviors. The total of 25,286 images was used to train and test the classification ResNet50 [29] network. In particular, 75% of images were for the training and 25% of them were for the test. The optimizer choice was Adam, and the model was trained for 150 epochs reaching a test accuracy of 99.98%.  Figure 12 shows the confusion matrices obtained for the training and test.

### 4.3. Semantic Localization System Experimentation

As aforementioned, the objective of this module is to help the robot get to know the location of places in houses. With this, the robot will be able to identify the place where it is.

In order to train the base model, we took video sequences from different residences and then randomly shuffled and distributed them into 70% training and 30% test splits.  Table 3 shows the final number of samples per category. We use only RGB frames.

**Figure 13:**
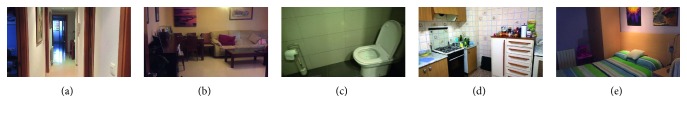
Sample images for each category of our home dataset: (a) corridor; (b) living room; (c) bathroom; (d) kitchen; (e) bedroom.

The experimentation described in this section was carried out using our own dataset which provides a semantic category for each RGB image. It is important to state that the base model was built with images from four different residences. The categories come from the location in which the images were acquired.  Figure 13 shows representative images for the 5 categories available in the dataset.

The experiments consist of measuring the performance of an already trained model with images from the house presented in Figure 9. It is worth noting that the cited model was trained on the dataset we described earlier.

For experiments in which new knowledge was included, we used images that were captured in the abovementioned house. In this house, we have the same semantic categories but different visual appearances. The robot then proceeded to capture new information about the environment that the system had failed to identify. Subsequently, the new information was added to the current learned model.

**Figure 14:**
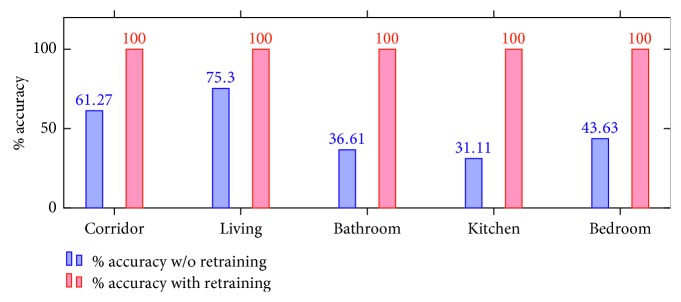
Accuracy of the SLS before and after applying the retraining process.

First, we comment on the experiments carried out in the different rooms using only the base model, and then we discuss what happened when the system had flaws in the classification and we capture information from the new environment. A summary of the results for the experiments performed can be found in Figure 14.

Experiment 1 establishes the baseline we use to compare the following experiments. The total accuracy of the test is 94.91%. This represents the starting line, as no new knowledge was added.

Experiments from 2 to 6 were performed in abovementioned house, obtaining results of (corridor ⟶61.27%), (living ⟶75.30%), (bathroom ⟶36.61%), (kitchen ⟶60.54%), and (bedroom ⟶43.63%) when the robot did not know the environment and 100% in all the places once it had added information about these places.

The experimentation confirms the accuracy of the system and validates it for its deployment for semantic localization uses.

### 4.4. Motion Planning System Experimentation

**Figure 15:**
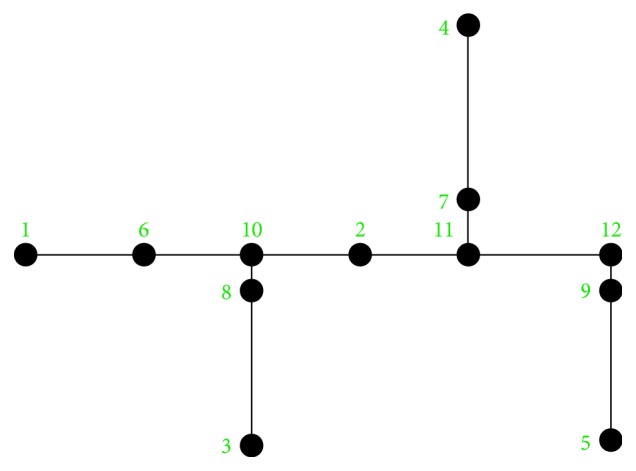
Numbered nodes of the house's graph representation.

For the experimentation of the motion planning system, we are using the concrete example of the house described in Figure 9, with the numerated nodes shown in Figure 15. We have defined the node types of every point in Sourcecode 3, so that every line number between 1 and 12 corresponds to the definition of the same numerated node. We have defined the connection between nodes in Sourcecode 4 with the associated cost and the direction that the robot must take to go from node *A* to *B*. As stated in Section 3.7, the definition of the connection between nodes is only made one-sided.

First of all, we calculate the paths from the kitchen to the others rooms, covering all the existing nodes in the graph. The results indicate that every node can be reached. To ensure the reliability of the rules that grant reverse connections, we calculate the reverse paths of the previous queries too.

Once we have checked that this system calculates all the paths and their reverses, we test the functionality of the dynamic predicate closed/1, so we cannot reach a goal following a path if there is some door closed.

We have covered all the possible paths between nodes in our experimentation. Due to space constraints, we only show as example the Execution Result 1.

### 4.5. Navigation and Mapping Experimentation

**Figure 16:**
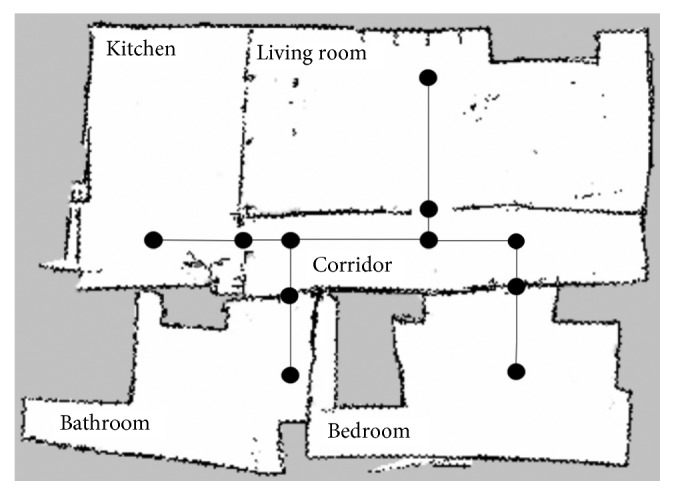
The image shows the actual map generated for the MPS with the correspondent graph superimposed. The room names correspond to the semantic labels used by the SLS. The map was iteratively generated by gmapping.

As stated in Section 3.8, we have used the *gmapping* algorithm in order to build the 2D static map of the environment, using a fake laser read from the depth sensor of Pepper. The results are shown in Figure 16. Additionally to the generated map, we have defined the position of the MPS nodes in order to associate a physical location to the semantic ones, so the robot can perform the navigation between the nodes.

**Figure 17:**
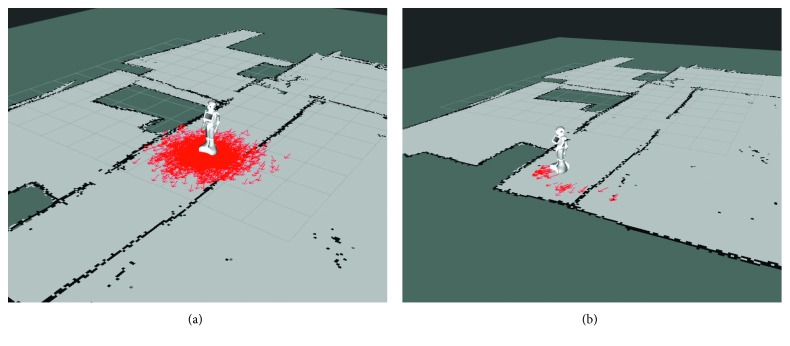
Adaptive Monte Carlo localization running. Every arrow represents a particle with an estimated 2D pose of the robot. (a) High uncertainty so there are multiple plausible poses of the robot (depicted as a big cloud of red arrows around the robot). (b) The robot saw a feature that helped to reduce the uncertainty, so the plausible poses are significantly reduced (shown as small clusters of red arrows around the robot).

Using this map, Pepper can perform the localization using the aforementioned *adaptive Monte Carlo localization* with its laser reads. As depicted in Figure 17, we can see the particles sampled by this algorithm with the most probable poses of the robot. When a well-identifiable location is captured by the laser, the density of the particles concentrates over its actual position.

The navigation has been successfully performed with the ROS *move_base* package. The costmaps generated by the planners according to the laser reads locate the dynamic obstacles and let them to compute the optimal path between the current pose and the goal. Additionally, Pepper incorporates an extra level of collision avoiding that blocks the movement of its base when the sonar detects an obstacle. This security margin from the Pepper-integrated system makes door crossing difficult when the door is not quite big.

### 4.6. People Tracking and following Behavior Experimentation

**Figure 18:**
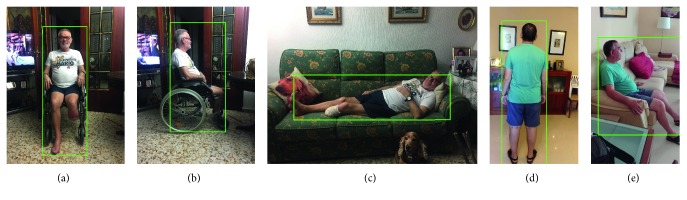
Some samples of the architecture performance, which is part of the people tracking subsystem. The robustness of the architecture is critical given the depicted cases, since the patients are highly likely to often render these poses.

In this case, the architecture of choice was not trained from scratch, but we adopted an already trained model. This model was trained on the COCO MS dataset which is able to accurately detect persons among other objects. The detection of the rest of the objects is ignored, so we only retrieve the detection of the label person. This model is accurate enough to detect persons in a variety of poses, even when the person is facing backwards the camera, sitting or lying in a bed or couch, or even if they are using a wheelchair as depicted in Figure 18. The accuracy of the architecture in these cases is specially important for our system because the patients are highly likely to often render these poses.

**Figure 19:**
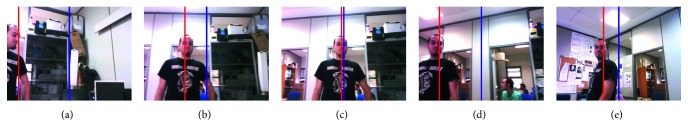
The robot tracks the person and always have him in the center of its sight by moving its base. The vertical blue line shows the center of the sight of the robot, whilst the red one depicts the center of the person. Both lines are aligned within a threshold.

The tracking method tries to maintain the patient always in the center of the robot's sight within a threshold and a preset distance. In the experiments, the centering threshold was set to 70 px. This threshold enables a fine centering process while avoiding excessive movement of the robot due to little displacements of the patient or flickering in the detected area of the person.  Figure 19 depicts the people tracking method. The clear space between the patient and the robot was set to 70 cm, which places the robot far enough to enable the free movement of both robot and patient, while being closer enough to assure a fluent interaction. There is also a 10 cm threshold for the same reason we mentioned earlier. The speed of the linear movement is set to 0.3 m/s and the speed of self-rotation to 0.3 rad/s. We noticed that this method is highly dependent of the response time. In our test setup, the mean image acquisition time is 126 ms, whilst the person detection takes 301 ms mean. Both measures include the intercommunication overhead.

The people tracking and following behavior performed robustly. The robot only lost track of the patient when he moved unusually fast, so it completely fell out of sight of the robot. In this case, the robot asked the patient to position himself in front of it, and the tracking resumed properly.

## 5. Conclusions and Limitations

A robotic system for monitoring and interacting with people affected by cognitive diseases is proposed in this paper. The system successfully integrates object recognition, activity recognition, localization, and navigation methods to remember and help the patients to perform their daily tasks.

Nonetheless, the system has some limitations. First, the initial stage where the map is created and the models are trained is mandatory and must be carried out by experts. In addition, the models for ORE and BRE have to be rebuilt if new objects are required, as they are needed for so far unconsidered tasks that we would like to add to the patient's schedule. This issue could be mitigated by creating a proper plan that considers the long-term evolution of the patient on the first place.

The system was supervised by ADACEA, which is a foundation for the acquired brain injured people, that ensured it effectively may help the patients to improve their self-autonomy and quality of life.

Finally, it is worth noting that there is a video in the supplementary materials that depict the different subsystems running in test environments.

## Figures and Tables

**Algorithm 1:**
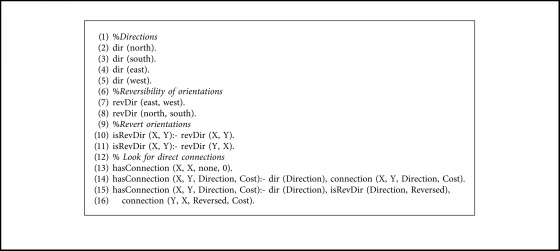
Definition of connection rules.

**Algorithm 2:**
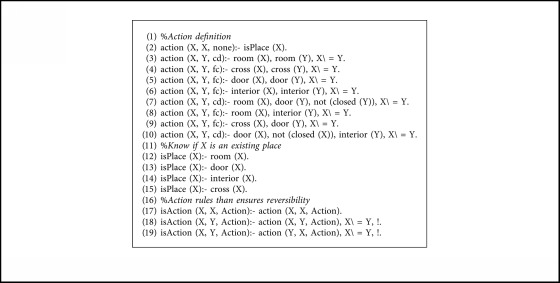
Definition of action rules.

**Algorithm 3:**
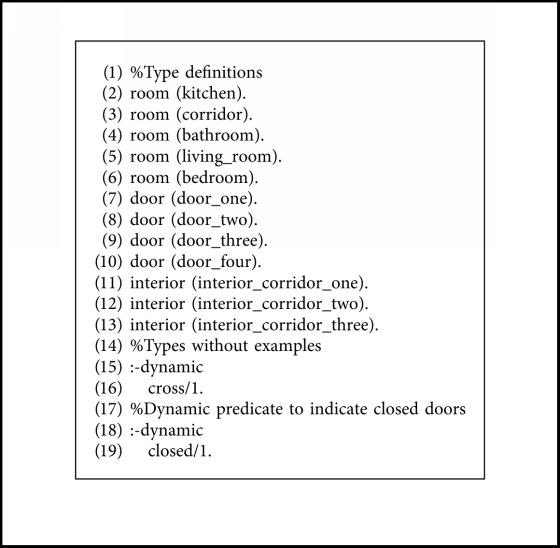
Definition of node types facts.

**Algorithm 4:**
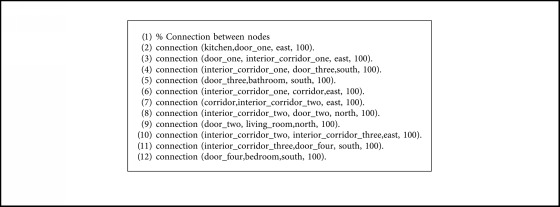
Definition of node connections facts.

**Algorithm 5:**
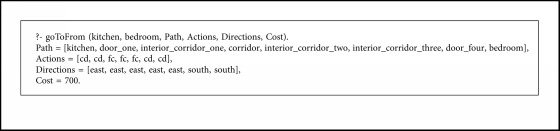
Execution result from kitchen to bedroom.

**Table 1:** Transition matrix between node types.

From/to	Room	Door	Cross	Interior
Room	cd	cd	ND	fc
Door	cd	fc	fc	cd
Cross	ND	fc	fc	ND
Interior	fc	cd	ND	fc

**Table 2:** Defined tasks for a test patient. Note that those tasks with no deadline in the scheduled column are contextual. The ORE goals define the objects that must be detected by the ORE. BRE goals define the behaviors that must be detected by the BRE. QRRE goals are the QR labels needed for that action.

Task	Location	Scheduled	ORE goals	BRE goals	QREE goals
Shave	Bathroom	—	Razor and bottle	Shave	—
Drink a glass of water	Kitchen	—	Glass and bottle	Pour	—
Brush teeth	Bathroom	9:30	Toothbrush and toothpaste	Brush teeth	—
Water the plant of the bedroom	Bedroom	10:30	Plant and water	Pour	—
Cook scrambled eggs	Kitchen	12:30	Eggs, skillet, and bowl	Pour and beat	—
Turn the AC on	Living room	—	Remote	—	—
Workout: rise the arms	Bedroom	15:00	—	Rise arm	—
Take a painkiller	Bathroom	15:30	—	—	Painkiller

**Table 3:** Images distribution per category.

Cat. ID	Category	Training	Test
1	Corridor	4,747	2,037
2	Living room	6,205	2,661
3	Bathroom	3,299	1,415
4	Kitchen	4,878	1,616
5	Bedroom	6,205	3,216

## Data Availability

The image data used to support the findings of this study have not been made available because they involve vulnerable social groups, and the persons in charge of them refuse the access to the data without a formal request.

## Supplementary Materials

Supplementary Materials Alongside the paper is attached a video demonstration of our proposal. In this video, the different pieces that integrate the system are depicted. First, the people tracking and following behavior is shown (Section 3.9). In this segment, a video from the point of view of the robot can be seen. The blue vertical line marks the center of the image, whilst the red one shows the center of the person, which is remarked in a green box. The characters in the left bottom corner represent the left, front, back, and right movement commands the system is issuing to the robot. Red is disabled, and green means the command is being sent. The user is wandering in a room, and the robot successfully tracks and follows the patient. The following segment shows the implementation of the programmed schedule and the context schedule. Both systems are explained in Section 3. The colors in the programmed schedule shows delayed or aborted tasks in red, imminent tasks in yellow, and the remaining tasks in blue. In this experiment, the localizations are being simulated by shuffling different locations. This is done to show the contextual schedule. Finally, two complete tasks are shown. First, a “take medicine A” task is due, so the robot asks the patient if he wants to attend the task. The user answers “OK,” so the robot uses its speech recognition capabilities (Section 3.5) to start the task. Then, the robot asks the patient to show the “medicine A.” In this case, the QRRE is used (Section 3.4) to properly recognize the QR code of that medicine. The second task is “pour a glass of water.” Once again the robot announces the task is due, and the patient triggers the task by answering “yes.” The robot asks the user to show a glass and the water, which are being recognized properly by the ORE (Section 3.2). Then, the pour action is detected using the BRE (Section 3.3). The detection scores are superimposed in the top left corner. This video feed comes from the camera of the robot. Note that the robot detects that it is not the proper room to perform the task (kitchen), but the navigation (Section 3.8) is disabled as the testing environment does not feature a kitchen.

## References

[1] Rooney C. O’., Hare Greg M. P. O., Donoghue R. D., Brian R. (1999). *What is a Social Robot*.

[2] Ikeda S., Arai F., Fukuda T. Vitro patient-tailored anatomical model of cerebral artery for evaluating medical robots and systems for intravascular neurosurgery.

[3] Ikeda S., Arai F., Fukuda T., Negoro M., Irie K., Takahashi I. Patient-specific neurovascular simulator for evaluating the performance of medical robots and instrumens.

[4] Kouroupetroglou C., Casey D., Raciti M. Interacting with dementia: the mario approach.

[5] Moyle W., Jones C., Sung B. (2016). What effect does an animal robot called cuddler have on the engagement and emotional response of older people with dementia? A pilot feasibility study. *International Journal of Social Robotics*.

[6] Rentschler A. J., Cooper R. A., Blasch B., Boninger M. L. (2003). Intelligent walkers for the elderly: performance and safety testing of va-pamaid robotic walker. *Journal of Rehabilitation Research and Development*.

[7] Machiel Van der Loos H. F., Joseph Wagner J., Smaby N. Provar assistive robot system architecture.

[8] Hsu P. E., Hsu Y. L., Chang K. W., Geiser C. (2012). Mobility assistance design of the intelligent robotic wheelchair. *International Journal of Advanced Robotic Systems*.

[9] Hillman M., Hagan K., Hagan S., Jepson J., Orpwood R. (2002). The weston wheelchair mounted assistive robot–the design story. *Robotica*.

[10] Falcone E., Gockley R., Porter E., Nourbakhsh I. (2003). The personal rover project: the comprehensive design of a domestic personal robot. *Robotics and Autonomous Systems Socially Interactive Robots*.

[11] Pineau J., Montemerlo M., Pollack M., Roy N., Thrun S. (2003). Towards robotic assistants in nursing homes: challenges and results. *Robotics and Autonomous Systems*.

[12] Pollack M. E., Brown L., Colbry D. (2003). Autominder: an intelligent cognitive orthotic system for people with memory impairment. *Robotics and Autonomous Systems*.

[13] Karlsson M., Engelbrektsson P., Hunter H., O’Niell A. M., Petrie H., Zoldan D. PAM-AID. personal adaptive mobility aid for the frail and elderly visually impaired. d3. 1. user requirement study.

[14] Fasola J., Mataric M. J. Robot exercise instructor: a socially assistive robot system to monitor and encourage physical exercise for the elderly.

[15] Srinivasa S., Ferguson D., Helfrich C. (2010). Herb: a home exploring robotic butler. *Autonomous Robots*.

[16] Wang T., Zhang H., Ma X., Zhu Y., Zhou Z., Qian B., Zeng D. (2011). A home nursing robot system. *Future Intelligent Information Systems*.

[17] Huete A. J., Victores J. G., Martinez S., Gimenez A., Balaguer C. (2012). Personal autonomy rehabilitation in home environments by a portable assistive robot. *IEEE Transactions on Systems, Man, and Cybernetics, Part C (Applications and Reviews)*.

[18] Srinivasa S. S., Berenson D., Cakmak M. (2012). Herb 2.0: lessons learned from developing a mobile manipulator for the home. *Proceedings of the IEEE*.

[19] Louie W. G., Vaquero T., Nejat G., Beck J. C. An autonomous assistive robot for planning, scheduling and facilitating multi-user activities.

[20] Ding J., Lim Y., Solano M. Giving patients a lift–the robotic nursing assistant (rona).

[21] Görer B., Ali Salah A., Levent Akin H. (2017). An autonomous robotic exercise tutor for elderly people. *Autonomous Robots*.

[22] Lv P., Zhou B., Wu K. (2018). A new remote health-care system based on moving robot intended for the elderly at home. *Journal of Healthcare Engineering*.

[23] Costa A., Martinez-Martin E., Cazorla M., Julian V. (2018). Pharos–physical assistant robot system. *Sensors*.

[24] Szegedy C., Ioffe S., Vanhoucke V., Alemi A. (2016). Inception-v4, inception-ResNet and the impact of residual connections on learning. http://arxiv.org/abs/1602.07261.

[25] Russakovsky O., Jia D., Su H. (2015). ImageNet large scale visual recognition challenge. *International Journal of Computer Vision (IJCV)*.

[26] Cao Z., Simon T., Wei S.-E., Sheikh Y. Realtime multi-person 2d pose estimation using part affinity fields.

[27] Simon T., Joo H., Matthews I., Sheikh Y. Hand keypoint detection in single images using multiview bootstrapping.

[28] Wei S.-E., Ramakrishna V., Kanade T., Sheikh Y. Convolutional pose machines.

[29] He K., Zhang X., Ren S., Sun J. Deep residual learning for image recognition.

[30] Brown J. (2018). *Zbar Bar Code Reader*.

[31] Saman G., Gilani S. A. M. Object recognition by modified scale invariant feature transform.

[32] Lowe D. G. (2004). Distinctive image features from scale-invariant keypoints. *International Journal of Computer Vision*.

[33] Rublee E., Rabaud V., Konolige K., Orb G. B. An efficient alternative to sift or surf.

[34] Rudinac M., Lenseigne B., Pieter P., Jonker Keypoint extraction and selection for object recognition.

[35] Aldebaran, http://doc.aldebaran.com/2-5/naoqi/audio/alspeechrecognition.html, 2018

[36] Cruz E., Rangel J. C., Gomez-Donoso F., Bauer Z., Cazorla M., Garcia-Rodriguez J. Finding the place: how to train and use convolutional neural networks for a dynamically learning robot.

[37] Annoy, https://github.com/spotify/annoy, 2018

[38] Grisetti G., Stachniss C., Burgard W. (2007). Improved techniques for grid mapping with rao-blackwellized particle filters. *IEEE Transactions on Robotics*.

[39] Perera V., Pereira T., Connell J., Manuela M., Veloso M. M. (2017). *Setting Up Pepper for Autonomous Navigation and Personalized Interaction with Users, CoRR, abs/1704*.

[40] Fox D., Burgard W., Frank D., Thrun S. Monte carlo localization: efficient position estimation for mobile robots.

[41] ROS (2018). *Move_Base*.

[42] Redmon J., Farhadi A. (2018). YOLOv3: an incremental improvement. http://arxiv.org/abs/1804.02767.

